# Formulation, Characterization and Cytotoxicity Effects of Novel Thymoquinone-PLGA-PF68 Nanoparticles

**DOI:** 10.3390/ijms22179420

**Published:** 2021-08-30

**Authors:** Nurul Shahfiza Noor, Noor Haida Mohd Kaus, Myron R. Szewczuk, Shahrul Bariyah Sahul Hamid

**Affiliations:** 1 Oncological and Radiological Sciences Cluster, Advanced Medical and Dental Institute, Universiti Sains Malaysia, Bertam, Kepala Batas 13200, Penang, Malaysia; nurulshahfiza@gmail.com; 2 Department of Physical Chemistry, School of Chemical Sciences, Universiti Sains Malaysia, Gulong 11800, Penang, Malaysia; noorhaida@usm.my; 3 Department of Biomedical and Molecular Sciences, Queen’s University, Kingston, ON K7L 3N6, Canada; szewczuk@queensu.ca

**Keywords:** TQ-PLGA-PF68, PLGA-PEG, Pluronics F68, emulsion–solvent evaporation, resistant breast cancer

## Abstract

Thymoquinone has anti-cancer properties. However, its application for clinical use is limited due to its volatile characteristics. The current study aims to develop a polymeric nanoformulation with PLGA-PEG and Pluronics F68 as encapsulants to conserve thymoquinone’s (TQ) biological activity before reaching the target sites. Synthesis of nanoparticles was successfully completed by encapsulating TQ with polymeric poly (D, L-lactide-co-glycolide)-block-poly (ethylene glycol) and Pluronics F68 (TQ-PLGA-PF68) using an emulsion–solvent evaporation technique. The size and encapsulation efficiency of TQ-PLGA-PF68 nanoparticles were 76.92 ± 27.38 nm and 94%, respectively. TQ released from these encapsulants showed a biphasic released pattern. Cytotoxicity activity showed that tamoxifen-resistant (TamR) MCF-7 breast cancer cells required a higher concentration of TQ-PLGA-PF68 nanoparticles than the parental MCF-7 cells to achieve IC_50_ (*p* < 0.05). The other two resistant subtypes (TamR UACC732 inflammatory breast carcinoma and paclitaxel-resistant (PacR) MDA-MB 231 triple-negative breast cell line) required a lower concentration of TQ-PLGA-PF68 nanoparticles compared to their respective parental cell lines (*p* < 0.05). These findings suggest that TQ encapsulation with PLGA-PEG and Pluronics F68 is a promising anti-cancer agent in mitigating breast cancer resistance to chemotherapeutics. In future studies, the anti-cancer activity of TQ-PLGA-PF68 with the standard chemotherapeutic drugs used for breast cancer treatment is recommended.

## 1. Introduction

Nanotechnology has been gaining research interest in recent years and evolved alongside the growing technological demand to improve therapy [1,2]. Current breast cancer therapy include surgery, radiotherapy, chemotherapy and hormone therapy [3]. Compared with present treatments for breast cancer, nanomedicine has many benefits. Nanomedicines are less prone to drug degradation while being transported with improved biocompatibility and increased delivery of drugs to tissues. Nanomedicine also exhibits great potential to effectively target and eliminate breast cancer stem cells involved in resistance.

Polymer nanoparticles have been developed for an effective drug-delivery of hydrophobic drugs and hormone regulators (taxanes, camptothecin, cisplatin and tamoxifen) [4]. Polymeric nanoparticles are core–shell structures, including polymeric micelles, capsules, colloids and dendrimers. Polymeric nanoparticles can be self-assembled in an aqueous solution using poly(ethylene glycol) (PEG) with amphiphilic and biodegradable polymers to avoid particle agglomeration [5]. The US Food and Drug Administration (FDA) and European Medicines Agency (EMA) approved the use of biodegradable and biocompatible polymers. These polymers include poly(lactic acid) (PLA), poly(D,L-lactide-co-glycolide) acid (PLGA) and poly(caprolactone) (PCL) [6]. Furthermore, the use of PLGA has gained attention for clinical application [7]. The PLGA polymers exhibit physical stability and better biocompatibility for the delivery of drugs, proteins and macromolecules (DNA, RNA and peptides) [8]. 

**Figure 1:**
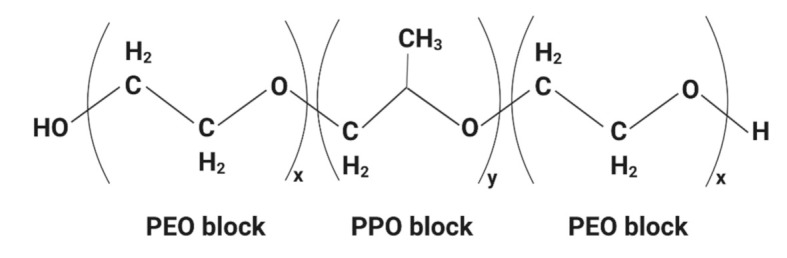
A general chemical structure of poloxamer. The image was created with BioRender.com.

Another essential component in the formulation of nanoparticles is poloxamers (Pluronics^®^). Poloxamers are made from the unique properties of non-ionic synthetic triblock copolymers with amphiphilic characteristics in the presence of hydrophilic poly(ethylene oxide) (PEO) block (x) and hydrophobic poly(propylene oxide) (PPO) block (y) (Figure 1) [9,10]. These hydrophilic and hydrophobic segments enable the self-assembly of one molecule to form unimolecular micelles with a hydrophobic inner core and hydrophilic outer shell in aqueous media [11,12]. These unimolecular micelles can encapsulate hydrophobic drugs with a high drug load [13]. The other benefits include the ease of preparation, improved solubility of the drugs, decreased toxicity, enhanced circulation, improved tissue penetration and most importantly, its biocompatibility with the biological system. The most commonly used poloxamers are poloxamer 188 (Pluronics^®^ 68) and poloxamer 407 (Pluronics^®^ 127) [9]. These two poloxamers are FDA-approved and listed in the US and European Pharmacopoeia [14]. They are non-toxic and non-irritant. Hence, they can act as a solubilizer, emulsifier, stabilizer and administer via oral, parenteral and topical routes [14]. In vivo studies reported paclitaxel-loaded Pluronics F68 exhibited higher anti-cancer efficacy than paclitaxel formulated in Cremophor EL [15]. Another study indicated that the docetaxel-loaded poly (ε-caprolactone)/Pluronics F68 achieved significant cytotoxicity in the docetaxel-resistance human breast cancer cell line [16]. Additionally, doxorubicin-loaded Pluronics F68-coated PLGA nanoparticles exhibited an antitumor effect against the experimental orthotopic 101.8 glioblastomas in rats upon intravenous administration [17]. Previous studies have evaluated the synthesis of PLGA with many different poloxamers, including Pluronics F68 [18,19]. Therefore, the formulation of PLGA-PEG and Pluronics F68 for the design of polymer nanoparticles can be suitable for the activity enhancement of the encapsulated drug. 

Plant-derived compounds for the treatment of breast cancer can be incorporated into nanoparticles. Among the natural compounds widely studied is thymoquinone (TQ), the chemical composition of the volatile oil from *Nigela sativa* seed extract. TQ exhibits pharmacological characteristics such as antioxidants, anti-inflammatory and anti-cancer activity [20]. Experimentally, it inhibits cancer cell growth and progression, based on the in vitro and in vivo study models [21,22]. Although the roles of TQ have been extensively studied, there are some limitations for its application due to its low solubility and fast degradation in an aqueous environment. As a consequence, the efficacy of the anti-cancer activity of TQ is reduced. Thus, encapsulation of TQ using nanotechnology will retain its bio-functionality, increasing TQ’s solubility in an aqueous environment and preventing degradation before reaching the target site. Another advantage is the nanoscale size of the formulation, enabling its penetration into the cancer cells. However, there is limited information regarding the application of polymeric TQ to treat drug-resistance breast cancers. Ganea et al. (2010) first described the encapsulation of TQ with PLGA [23]. This study showed that TQ-loaded PLGA has an anti-proliferative effect on breast cancer cell lines. A similar outcome was also reported by several studies using various nanoplatforms to improve the efficacy of TQ. In the present study, the TQ compound was incorporated into polymeric PLGA-PEG and Pluronics F68 using the emulsion–solvent evaporation technique [24]. This method is used mainly to prepare water-insoluble compounds to obtain homogeneously sized nanoparticles with higher encapsulation efficiency. The study aimed to develop and characterize the novel TQ-loaded PLGA-PEG nanoparticles in the presence of Pluronics F68. It is a proof-of-concept study to demonstrate its therapeutic effects in tamoxifen-resistance human breast cancer cells. 

## 2. Results

All subsequent analyses and results presented here were acquired from TQ-PLGA-PF68 nanoparticles stored after freeze-drying. The samples were characterized using Fourier Transform Infrared Spectroscopy (FTIR) study, Dynamic Light Scattering (DLS) technique, transmission electron microscopes (TEM), entrapment efficiency, drug release profiles and cell cytotoxicity analyses. These analyses were investigated because the interaction between nanomaterials and biological targets could significantly affect the physiological interactions.

### 2.1. Characterization of TQ-PLGA-PF68 Nanoparticles 

#### 2.1.1. FTIR Spectroscopic Analysis

FTIR spectral analysis of TQ, PLGA-PEG and Pluronics F68 vibrational modes in their native form and nanoparticle formulations provides insight into the structural integrity and retention of functional groups in the nanoparticles formed. The infrared spectra ranged from 4000 to 600 cm^−1^ of TQ-PLGA-PF68 nanoparticles, PLGA-PEG, Pluronics F68 and TQ and are summarized in Table 1. TQ-PLGA-PF68 nanoparticles had slightly moved peaks either to a higher or lower wavelength corresponding to the native spectra of PLGA-PEG or Pluronics F68. Some peaks were not found in nanoparticles, even though present in individual components. Notably, there were no TQ absorption bands detected. These results indicate that the TQ was successfully conjugated with the PLGA-PEG polymer and Pluronics F68 in forming TQ-PLGA-PF68 nanoparticles.

#### 2.1.2. Polydispersity Index (PDI) and Zeta Potential

PDI and zeta potential values of TQ-PLGA-PF68 nanoparticles were evaluated using the dynamic light scattering (DLS) technique. The PDI value was 0.23 ± 0.01, which is within the range of acceptable PDI values for the drug to be transported through biological barriers in the body. In the current study, TQ-PLGA-PF68 nanoparticles had a zeta potential of −19.83 ± 0.69 mV. This result suggests better stability of the TQ-PLGA-PF68 nanoparticles as zeta potential with a higher negative magnitude value representing the nanoparticle’s stability. It can be attributed to the presence of Pluronics F68 that is associated with improved stability of nanoparticles [25] 

#### 2.1.3. Transmission Electron Microscope (TEM)

**Figure 2:**
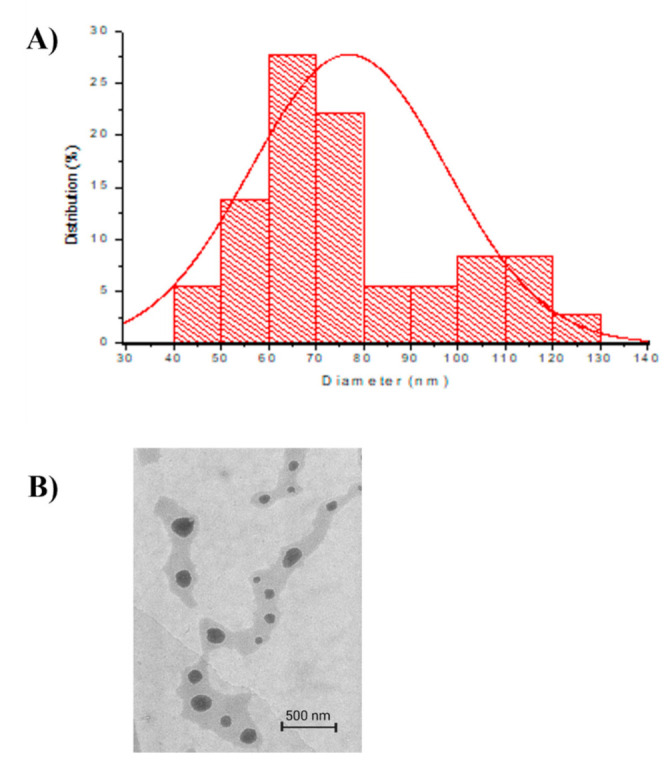
Particle size distribution and particle structure of TQ-PLGA-PF68 nanoparticles. (**A**) Particle size distribution of TQ-PLGA-PF68 nanoparticles with the average size at 76.92 ± 27.38 nm, processed by Microcal Origin 6 software (Origin Lab, United States); (**B**) TQ-PLGA-PF68 nanoparticle image observation at 10,000× magnification using FEI CM12 transmission electron microscopy.

**Figure 3:**
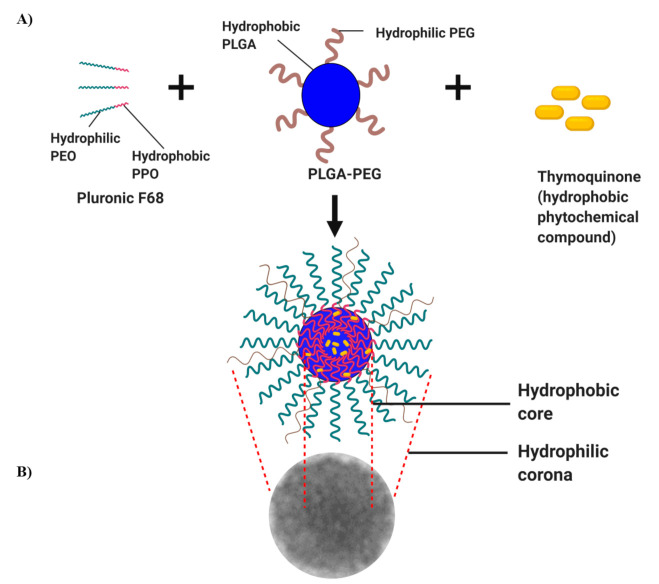
Schematic diagram depicting TQ nanoparticle formulations by emulsion–solvent evaporation technique. (**A**) TQ self-assembles with an amphiphilic molecule of Pluronic F68 and PLGA-PEG, forming hydrophobic core while intact TQ is located within the core. (**B**) TEM observation of TQ-PLGA-PF68 nanoparticles. The image was created with BioRender.com.

The shape of polymeric TQ-PLGA-PF68 nanoparticles was measured using TEM. TQ-PLGA-PF68 nanoparticles size was 76.92 ± 20.64 nm, and the spherical shape is depicted in Figure 2. The value of zeta potential could influence this observation. The farther the zeta potential deviates from 0 mV, the smaller the particle size. According to previous studies, nanoparticles smaller than 100 nm and spherical improved optimal anti-cancer efficacy with minimal side effects [26]. Figure 3 shows that the outer layer of the TQ-PLGA-PF68 nanoparticles was lighter than the core area. This observation suggests that the two physicochemical constituents were present within the nanoparticles at the shell and core.

### 2.2. Encapsulation Efficiency (EE) of TQ-PLGA-PF68 Nanoparticles

It was observed that TQ was successfully encapsulated into PLGA-PEG and Pluronic F68 with an EE of 94.47%. This result indicates that only a small amount of TQ was portioned out throughout the encapsulation processes. High entrapment efficiency for hydrophobic compounds (80–98%) with PLGA-PEG and Pluronics as encapsulants are usually observed using the emulsion–evaporation method [27].

### 2.3. Effect of pH of the Release Medium on In Vitro TQ Release

**Figure 4:**
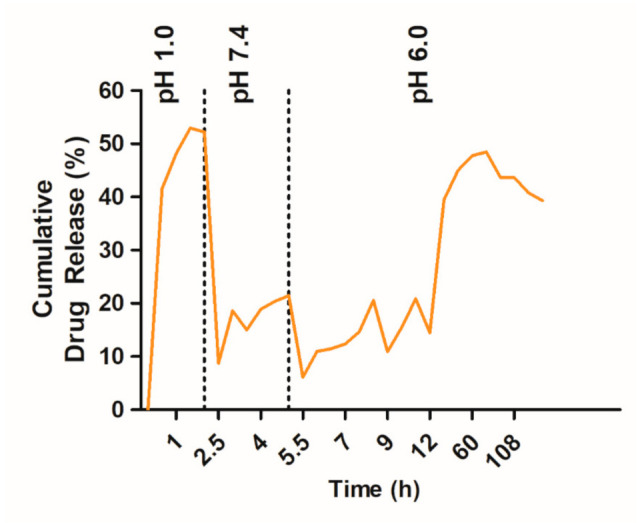
Release profiles of TQ-PLGA-PF68 nanoparticles formulation in the simulated gastrointestinal tract.

The release of TQ was studied as a function of pH to simulate the gastrointestinal tract (GIT) microenvironment. At pH 1.0 (simulated gastric fluid in the stomach), the in vitro cumulative TQ release profile with an initial burst release was 52% at 2 h (Figure 4). This result was due to the rapid dispersion of TQ present on the surface of the PLGA. However, pH 7.4 represents a condition similar to the small intestine. There was slow release at a constant rate of up to 21%, as the entrapped TQ slowly diffused into the medium. Later, a low burst release of TQ (48%) was observed in the medium at pH 6.0 and subsequently decreased. Here, a long intestinal transit time was observed to ensure the complete release of entrapped TQ. The dual responses to both acidic and reducing environments could efficiently release the entrapped TQ. Moreover, these dual responses to the simulated GIT fluids suggest that the PLGA-PEG and Pluronics F68 are potential carriers for TQ delivery. The biphasic release pattern was similar to those reported previously [28] and is a common characteristic of the encapsulated polymeric nanoparticles.

### 2.4. Titration of Drug Sensitivity on Drug-Resistant Cell Lines

Generally, the results revealed that the resistant subtypes, namely, tamoxifen-resistant (TamR) MCF-7, TamR UACC 732 and paclitaxel-resistant (PacR) MDA-MB 231 breast cancer cell lines exhibited a higher IC_50_ value than their parental cells. The IC_50_ values and resistant index (RI) of each cell line for TamR and PacR cells are in Table 2. TamR MCF-7 IC_50_ value was higher than parental MCF-7 (MCF-7-P) (29.80 ± 5.16 versus 12.83 ± 6.41; *p* < 0.05). The IC_50_ value of PacR MDA-MB 231 was greater than the parental cells (34.11 ± 8.00 versus 21.48 ± 3.71; *p* < 0.05), and IC_50_ TamR UACC 732 was greater than parental UACC 732 (UACC 732-P) (26.29 ± 6.44 versus 19.80 ± 0.33; *p* < 0.05). The resistant index (RI), which reflects drug resistance potential for TamR MCF-7, was 2.32-fold more resistant to tamoxifen. PacR MDA-MB 231 was 1.59-fold more resistant to paclitaxel, and TamR UACC 732 was 1.32-fold more resistant to tamoxifen compared to their respective parental cell lines. The results indicated a relatively low level of resistance to the drugs given. Despite the low resistance level, the results could still be denoted as in the clinically relevant range, 1.5–5-fold increase from the parental cell line’s IC_50_ value [29].

### 2.5. In Vitro Cytotoxicity Study

**Figure 5:**
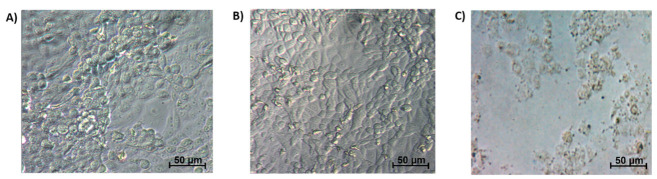
Representative images of phase contrast microscopic observation of TQ-PLGA-PF68 nanoparticles and the encapsulant (Pluronics and PLGA-PEG) cytotoxic effect on the resistant breast cancer cell lines. (**A**) 2% (*w/v*) Pluronics F68; (**B**) 2.5% (*w/v*) PLGA-PEG; (**C**) 96.34 µg/mL TQ-PLGA-PF68 nanoparticles. Cells were observed at 100× magnification.

The IC_50_ value for each of the cell lines with respective treatments is summarized in Table 3. TamR MCF-7 cells required a higher concentration of TQ-PLGA-PF68 nanoparticles than MCF-7 to achieve IC_50_ (*p* < 0.05). The other two resistant subtypes (TamR UACC 732 and PacR MDA-MB 231) required a lower concentration of TQ-PLGA-PF68 nanoparticles compared to their respective parental cell lines (*p* < 0.05). Figure 5 represents the images of the cytotoxic effect of Pluronics F68, PLGA-PEG and TQ-PLGA-PF68 nanoparticles on the resistant breast cancer cell lines. 

## 3. Discussion

Nanomedicine has emerged as a new technology in the development of the drug-delivery. This technology is ideal for improved permeability and retention in solid tumors [32]. It generates particles with a nanosize range between 100 nm and 300 nm. These smaller size particles can reduce sedimentation rate and improve physical stability.

In the present study, the TQ compound was encapsulated with polymeric PLGA-PEG and Pluronics F68 by a emulsion–solvent evaporation technique. This technique is the most common method used to encapsulate hydrophobic compounds [33]. TQ was dissolved in dichloromethane (DCM), incorporated with polymeric PLGA-PEG and dispersed in Pluronics F68 aqueous solution. The dispersion was essential to control the nucleation process and enhanced molecular diffusion. The emulsion was homogenized to form a nano-emulsion. The DCM content in nanosuspension was evaporated to leave behind the TQ-PLGA-PF68 nanoparticles. The emulsion method has better performance for encapsulation efficiency compared with other preparation methods. The formulation of PLGA-PEG and Pluronics F68 as excipient was completed to improve the stability and effectiveness of polymeric PLGA-PEG. A recent study stated that donepezil incorporated into PLGA in the presence of Pluronics F68 caused a significant dose-dependent decrease in both gene and protein expression levels of IL-1β, IL-6, GM-CSF and TNF-α in Alzheimer’s disease [34]. However, to date, little is known on the effects of encapsulated TQ with polymeric PLGA-PEG and Pluronics F68 to treat chemoresistant breast cancer cells. Therefore, the present investigation aimed to perform the encapsulation and characterization of TQ-PLGA-PF68 nanoparticles to assess their effect on chemoresistant breast cancer cells.

The interaction between the functional groups of TQ with the nanosuspension was confirmed with FTIR spectral analysis. The vibrational modes indicate the extent of structural integrity and the retentional functional group in TQ nanoparticles (Table 1). Pure TQ exhibited a strong vibrational peak at 1645 cm^−1^ that represents –C=O group vibration. After the formation of nanoparticles, there was a shift of the carbonyl group to 1751 cm^−1^. This result could be contributed by the confinement of nanoparticulate TQ within the suspension that limits the keto-enol tautomerism. The intense band at 2967 cm^−1^ represents the C–H stretching of the aliphatic group. However, this peak shifted to 2882 cm^−1^ after the formation of nanoparticles. This shifting movement can be due to the interaction of aliphatic chains with the inner hydrophobic core of nanosuspension. The analysis confirmed that TQ was entrapped within the nanosuspension without further modification of TQ. The nature of the interaction between TQ and nanosuspension was a hydrophobic and hydrogen bond interaction. This structure was concluded based upon the movement of vibration frequency that corresponds to the TQ functional groups. TQ encapsulated within the nanoparticle was therefore likely to maintain its functionality. TQ nanosuspension must sustain its activity for delivery to the target site with desired therapeutic action.

In the present study, TQ encapsulated into polymeric nanoparticles was performed to enhance its bioavailability and therapeutic efficiency. Accordingly, characteristics of polymeric TQ nanoparticles were evaluated based on the particle size, polydispersity index (PDI) and morphological features. The particle size and PDI of nanocarrier systems are the main physicochemical attributes to the maximum cellular uptake and therapeutic index and the bioavailability of the encapsulated therapeutic compound [35]. The observed particle size of TQ-PLGA-PF68 nanoparticles was in the nano range with less than 100 nm. Findings suggest that smaller nanoparticles will have a larger surface area that will enable better interaction with the target components [25] and are suitable for targeting tumor tissues [36]. Additionally, polymeric nanoparticles with sizes <200 nm would be efficiently taken up by cells [37]. Thus, data from this study showed that the synthesized TQ-PLGA-PF68 nanoparticles were at the optimal size for drug delivery and cellular uptake. 

PDI measures particle homogeneity that varies from the range of 0 (for a perfectly uniform sample for the particle size) to 1 (for a highly polydisperse sample with multiple particle size populations) [35]. The PDI value of TQ-PLGA-PF68 nanoparticles was 0.2, which indicates that particles of TQ-PLGA-PF68 nanoparticles were in monodispersity and homogeneous along with narrow size distribution. Values of 0.2 and below are acceptable in the application of polymer-based nanoparticle materials [38]. A PDI value close to zero indicates higher homology between the particles, suggesting the absence of nanoparticle aggregation. The presence of aggregation will hinder the proper targeting of nanoparticles to cells and tissues. It may reduce cellular uptake and lower cytotoxicity as aggregation causes an increase in particle size and reduce the surface area. Furthermore, aggregates will form sediments and will not be bioavailable [39]. Furthermore, adding Pluronics F68 to the nanoparticle formulation provided additional steric stability, preventing fine particles aggregation [16], thus resulting in smaller particle size and narrow size distribution.

In addition to size, the shape of nanoparticles is another critical parameter that can influence the circulation time, biodistribution, cellular uptake and targeting in cancer drug delivery [40]. TEM observation revealed the spherical shape of TQ-PLGA-PF68 nanoparticles. Biodistribution studies have demonstrated that the uptake of spherical nanoparticles is favored for drug delivery applications [27,29]. 

Zeta potential is the surface charge of a nanoparticle determined by its chemical properties at the coating or dispersing agent. The zeta potential significantly affects the various properties of nano-drug delivery systems, such as the nanoparticles’ stability, release rate in the bloodstream and absorption into body membranes [38]. The zeta potential value obtained in this study was −19.83 ± 0.69 mV, where the dispersion was expected to be stable. Generally, most of the biological cells have zeta potential in the range of −5 to −15 mV. This range indicates that the nanoparticles might interact through a receptor-mediated mechanism. The binding of nanoparticles happens when a robust receptor–ligand bond overcomes electrical repulsion [41]. Moreover, the negatively charged nanoparticles are cleared more slowly from the blood than positively charged after intravenous (IV) administration. They remained in the bloodstream for a longer time [42]. Moreover, negatively charged nanoparticles had lower cytotoxicity than positively charged nanoparticles and those approved by the FDA [38].

The nanoparticles were further characterized to determine their entrapment efficiency (EE). The EE of TQ-PLGA-PF68 nanoparticles was 94.47%. The higher EE obtained might be contributed by the combination of PLGA and polyethylene glycol (PEG) and the Pluronics F68 used. This finding was in line with the previous study recorded for EE of hydrophobic compounds encapsulated with polymeric PLGA such as eugenol (98%), trans-cinnamaldehyde (92%) [43] and α-tocopherol (95.4%) [44]. Besides, a previous study has recorded EE of TQ-based nanoparticles such as TQ-PLGA nanoparticles (62%), TQ-chitosan nanoparticles (63%) and TQ-liposome nanoparticles (90%) [22,40,41]. Moreover, high EE for hydrophobic compounds (80–98%) with PLGA as encapsulant were usually observed using the emulsion evaporation method [27]. For instance, etoposide-loaded nanoparticles were prepared to apply nanoprecipitation, and emulsion–solvent evaporation methods using PLGA in the presence of Pluronics F68 showed a high EE of around 80% [45]. Thus, in the present study, the current approach using the emulsion–solvent evaporation technique provided a successful TQ entrapment within the core of polymeric PLGA-PEG and Pluronics F68.

Formulation of TQ-PLGA-PF68 nanoparticles resulted in the typical biphasic release profiles that are associated with polymeric delivery systems. Results obtained indicated that the release rate of TQ from TQ-PLGA-PF68 was higher in an acidic environment compared to the physiological pH 7.4. A previous report by Fang et al. (2016) discovered curcumin release from F68-Cis-Cur micelles was almost 60% at pH 6.4 and 5.0, but only achieved 40% Cur at pH 7.4 after 96 h [46]. A similar finding was reported previously; the release rate of TQ was higher in acidic pH than in neutral pH [24]. The PLGA biodegradability property influenced this phenomenon. The PLGA tends to have fast de-structuration in an acidic environment with less lactate monomer produced, indicating less breaking of ester bonds. Meanwhile, in the primary pH condition, the degradation occurs slowly with the high production of lactate. Thus, it was clear that different pHs affected the TQ release from the TQ-PLGA-PF68 nanoparticles. 

Free TQ has been used in pre-clinical studies of arthritis, diabetes and hypercholesterolemia (https://clinicaltrials.gov/ct2/results?cond=&term=thymoquinone). It does not exhibit toxicity on normal cells [47] and is known for its eminent activity against many human carcinomas [48]. In the present study, the cell viability analysis was completed on parental and resistant subtypes of breast cancer cell lines as preliminary screening to assess the effectiveness and toxicity of the synthesized nanoparticles. The cells used were the ER-positive (MCF-7), HER2-positive (UACC 732) and triple-negative breast cancer cells (TNBC) (MDA-MB 231) and their resistant phenotypes (TamR MCF-7, TamR UACC 732 and PacR MDA-MB 231 cells). Analysis was completed with the MTS (Cell Proliferation; Colorimetric) assay to determine the viability of these cell lines. The IC_50_ value obtained for TamR MCF-7 cells was slightly higher than its parental MCF-7 cell line. An earlier study by Ahmad et al. (2020) indicated that the cytotoxicity of free TQ on TamR MCF-7 breast cancer cells was 8.25 μM [49]. This result suggests substantial cytotoxic effects as classified by National Cancer Institute and the Geran Protocol [50]. In the present study, the higher cytotoxicity value of TQ-PLGA-PF68 nanoparticles against TamR MCF-7 cells could be attributed to the nanoparticles’ physical and chemical properties that conserve their activity via a slow release. The IC_50_ values for TamR UACC 732 and in PacR MDA-MB231 cells were lower than their respective parental cell lines. These findings suggest that TamR UACC 732 and PacR MDA-MB 231 cells were more sensitive towards TQ-PLGA-PF68 nanoparticles compared to TamR MCF-7 cells, indicating that TQ-PLGA-PF68 nanoparticles exhibited a different effect on the subtypes of resistance breast cancer cells. The difference in effects of TQ-PLGA-PF68 nanoparticles could be due to the inhibition of different target proteins explicitly expressed in each subtype. This result would need to be further investigated. The current study did not demonstrate a cytotoxic effect of Pluronics F68 (2%, *w/v*) and PLGA-PEG (2.5%, *w/v*) as indicated by the morphological appearance of the cells studied. In contrast, there was a reduction in the cell number and size following treatment with TQ-PLGA-PF68 nanoparticles. This finding is supported by Al Khateb et al. (2016), whereby they reported that the nanoparticle formulation with 20% (*w/w*) of Pluronics 127 and Pluronics F68 was a non-toxic or irritant to the corneal mucosa. Hence, they may be suitable for application in ocular drug delivery [51]. Similarly, incorporation of ibuprofen with 15% (*w/w*) of Pluronics 127 did not exhibit cytotoxicity in Y-79 human retinoblastoma cells [52]. Taken together, the nanoparticle formulation with PLGA-PEG and Pluronics F68 may improve the unification of the nanoparticles into the membrane and the subsequent translocation into the cells, thus increasing the sensitization of the resistant breast cancer cells to TQ.

## 4. Materials and Methods

### 4.1. Synthesis of TQ-PLGA-PF68 Nanoparticles

TQ-PLGA-PF68 nanoparticles were prepared by encapsulating TQ (Sigma Aldrich, Germany) with polymeric polylactic acid/polyglycolic acid (PLGA) and polyethylene glycol (PEG)-5000 (PLGA-PEG) (Sigma Aldrich, Taufkirchen, Germany) using the emulsion–solvent evaporation method. This method is used to obtain homogeneously sized nanoparticles, particularly in the preparation of water-insoluble compounds. Fifty mg of PLGA-PEG was dissolved in 2 mL of dichloromethane (DCM) (Qrec, Chon Buri, Thailand) as an oil phase and left overnight to obtain a uniform solution. TQ compound (5 mg) was then added to the solution. The suspension was then sonicated for 2 min to produce solid/oil primary emulsion. Next, emulsification was completed by adding 20 mL of Pluronics F68 (molecular weight of 8500 Da) (Sigma Aldrich, Germany) in the aqueous phase. Pluronics F68 is a family of non-ionic surface-active agents. It is a block copolymer of hydrophobic propylene oxide sandwiched between two hydrophilic ethylene oxide blocks [53]. Here, Pluronics was used as a surfactant for the TQ-PLGA-PEG solution in DCM. It was stirred for 45 min at room temperature. The step is essential because it helps achieve the supersaturation state of the mixtures, enabling precipitation of ultra-fine amorphous nanoparticles. The mixture was vortexed for 10 s on a high setting. Subsequently, sonication was completed for 3 min to generate solid/oil/water (S/O/W). The next step was removing the organic solvent from the suspension with a rotary vacuum evaporator (Eyela, Tokyo, Japan) at 50 °C. Finally, the nanoparticle samples were centrifuged at 10,000× *g* for 20 min at 4 °C. Next, 2 mL of deionized water was added to the pellet that contained nanoparticles. It was dried in a lyophilized freeze-drier (Martin Christ Freeze Drier, Osterode am Harz, Germany) and stored at 4 °C before experiments. 

### 4.2. Characterization of TQ-PLGA-PF68 Nanoparticles

#### 4.2.1. FTIR Spectroscopic Analysis

TQ-PLGA-PF68 nanoparticles were probed with the FT-NIR spectrometer Frontier (PerkinElmer) to confirm the encapsulation of the TQ compound within the core structure of PLGA-PEG and Pluronics F68 molecules. In brief, the lyophilized TQ-PLGA-PF68 nanoparticles and pure substances (TQ, PLGA-PEG, Pluronics F68) were placed on the FT-NIR spectrometer Frontier sample compartment (Perkin Elmer, Boston, MA, USA) for the spectra acquisitions. Force was applied using the pressure arm to the sample, pushing it onto the crystal surface. The range of the scan covered was from 4000 to 600 cm^−1^. The acquired spectra were processed using Frontier’s Spectrum 10 software (Perkin Elmer, USA).

#### 4.2.2. Polydispersity Index (PDI) and Zeta Potential

Particle size distribution and zeta potential of TQ-PLGA-PF68 nanoparticles were determined using Malvern Zetasizer with Zetasizer Software 7.11 (Malvern Instruments, Malvern, UK) using dynamic light scattering (DLS) technique. Lyophilized TQ-PLGA-PF68 nanoparticles (1% *w/v*) were dissolved in deionized water. They were then vortexed and sonicated for 2 min. Each sample was measured in triplicates. The value of PDI may vary from 0.01 (monodispersed particles) to 0.5–0.7. A PDI value of more than 0.7 indicates a broad particle size distribution of the formulation and would tend to aggregate (In Supplementary Materials of [54]). Meanwhile, zeta potential values >+25 mV or <−25 mV have high degrees of particle stability [55].

#### 4.2.3. Transmission Electron Microscope (TEM)

Morphology of the TQ-PLGA-PF68 nanoparticles was observed using a transmission electron microscope (TEM), namely, FEI CM12 transmission electron microscopy (Thermo Fisher Scientific, Eugene, OR, USA). Images captured were later analyzed using the AnalySIS Docu version 3.2 image analysis system (Soft Imaging System, GmbH, Munster, Germany). Lyophilized TQ-PLGA-PF68 nanoparticles (1% *w/v*) were suspended in deionized water and vortexed for 1 min. TQ-PLGA-PF68 nanoparticles’ aqueous dispersion was then placed on a copper TEM grid. The excess amount of liquid was immediately removed by using filter paper. After that, nanoparticles were negatively stained with 2% phosphotungstic acid (PTA) and drained with filter paper before TEM observation. Images were captured, and particles in each quadrant were measured. Microcal Origin 6 software (Origin Lab, Northampton, MA, USA) was used to generate the TQ-PLGA-PF68 nanoparticles distribution histogram plot size.

### 4.3. Encapsulation Efficiency (EE) of TQ-PLGA-PF68 Nanoparticles

EE is the percentage of drug that is successfully entrapped into the nanoparticle and is regarded as a crucial step in characterizing the quality of the nanoformulations [56]. In the present study, TQ-PLGA-PF68 nanoparticles formulation was centrifuged at 30,000× *g* for 15 min. After centrifugation, the supernatant was removed, and 1 mL of ultrapure water was added to the pellet. Then, it was sonicated for 5 min to free the unencapsulated TQ. Next, centrifugation was completed at 30,000× *g* for 15 min. Finally, UV measurement was taken at 257 nm, which represents TQ absorption. The following equation was used to calculate the percentage of TQ EE:

(1) $$\mathrm{EE}\;\left(\%\right)=\frac{\mathrm{Amount}\;\mathrm{of}\;\mathrm{TQ}\;\mathrm{in}\;\mathrm{nanoparticle}\;\mathrm{formulation}\;}{\mathrm{Amount}\;\mathrm{of}\;\mathrm{initial}\;\mathrm{TQ}}\times 100$$

### 4.4. In Vitro Release of TQ from of TQ-PLGA-PF68 

TQ release was investigated in pH 1.0, pH 6.0 and pH 7.4 to simulate the gastrointestinal tract (GIT) pH conditions. Lyophilized TQ-PLGA-PF68 nanoparticles (10 mg) were placed in the dialysis tubing (10 K MWCO, Thermo Scientific, Waltham, MA, USA) and left to submerge in pH 1.0 (0.1 N HCl solution). Later, they were placed in pH 6.0 (PBS solution) and pH 7.4 (PBS solution). Samples were placed in a thermostatic rotary shaker with a speed of 100 rpm at 37 °C. Drug release was measured by sampling 1 mL of the outer media every 30 min intervals. An equal volume of fresh media was then added to replenish total media. The concentration of TQ released from TQ-PLGA-PF68 nanoparticles was measured with a UV spectrophotometer at 257 nm. Results are expressed as a percentage of the cumulative amount of TQ released versus time.

(2) $$\mathrm{Cumulative}\;\mathrm{drug}\;\mathrm{release}\;\left(\%\right)=\frac{\mathrm{Mt}}{M\theta }\times 100$$

Mt is the amount of drug released at time t, and M_θ_ is the initial drug in nanoparticle formulation. The drug release data were averaged based on three different measurements.

### 4.5. Cell Culture and Culture Conditions

The commercial human breast cancer cell lines MCF-7 (ATCC^®^ HTB-22™), UACC-732 (ATCC^®^ CRL-3166™) and MDA-MB-231 (ATCC^®^ HTB-26™) were used in the experiments. All cell lines were purchased from the American Type Cell Collection (ATCC) (Manassas, VA, USA). They were grown in RPMI 1640 medium with L-glutamine, 10% heat-inactivated fetal bovine serum (FBS) and 1% antibiotics (10,000 U/mL of penicillin, 10,000 µg/mL of streptomycin). Cells were grown in 25 cm^2^ flasks (Fisher Scientific, Corning, NY, USA) that contained 5 mL of complete growth medium and incubated at 37°C humidified incubator with 5% carbon dioxide (CO_2_). After 72 h of incubation, cells were detached from the flask with 0.05% trypsin (2.5 g/L trypsin, 1 mmol/l EDTA solution) and left in the incubator for 10 min prior for regular cell sub-culturing.

**Figure 6:**
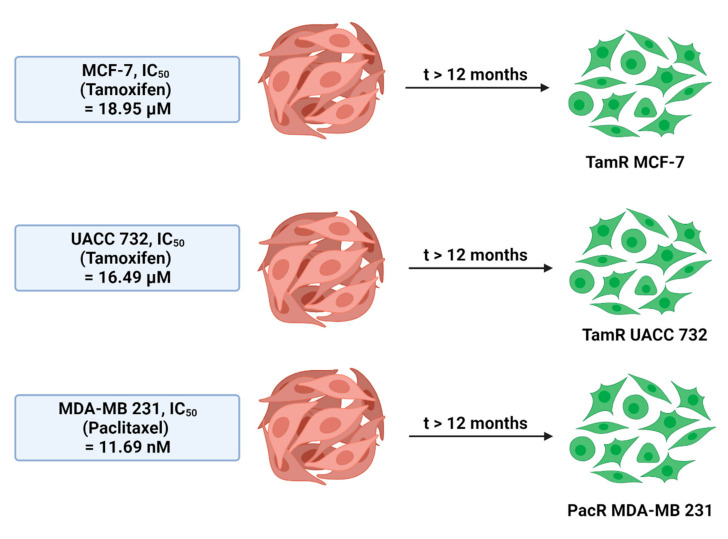
Overview of the generation of cell lines. The original cell lines were split, and new cell lines were generated parallel by treatment with a gradually increasing concentration of tamoxifen or paclitaxel. Starting dose for treatment of the cell line commenced at 10% of the chronic IC_50_ dose [57]. RI = IC_50_ of drug-resistant phenotypes/IC_50_ of parental cell line [30,31]. The image was created with BioRender.com.

To develop drug-resistant cell lines, two types of drugs were used to generate the resistant cell line models: tamoxifen and paclitaxel. MCF-7 and UACC 732 cell lines were treated with tamoxifen. Meanwhile, the MDA-MB-231 cell line was treated with paclitaxel. The model of drug-resistance cell lines was developed by using the pulse method. Initially, the parental cells were treated with 10% of the chronic IC_50_ dose to ensure cells growth adaptation before exposing to a higher drug dosage [57]. This experiment was performed for more than 1 year to ensure the cells develop into drug-resistance cell lines (Figure 6), followed by titration of drug sensitivity to confirm the resistant index (RI).

### 4.6. Cell Viability Assay

Cell viability assay was quantified using MTS assay kit, CellTiter96^®^ Aqueous Non-Radioactive Cell Proliferation Assay (Promega, Madison, WI, USA) to determine the 50% inhibitory concentration (IC_50_) of TQ-PLGA-PF68 nanoparticles effects on parental and resistant breast cancer cells. Measurement was taken at 490 nm. This test was conducted on different types of cell lines that included parental and drug-resistance breast cancer cell lines, namely, MCF-7, UACC 732, MDA-MB 231 and tamoxifen-resistant (TamR MCF-7 and TamR UACC 732) and paclitaxel-resistant (PacR MDA-MB 231) cell lines. The cells (1 × 10^4^) were plated in a 96-well plate and left overnight in a CO_2_ incubator to promote adhesion. A dose–response experiment was carried out with TQ-PLGA-PF68 nanoparticles range from 0–200 μg/mL for 72 h at 37 °C. The cells were treated with 2% (*w/v*) Pluronics F68 and 2.5% (*w/v*) PLGA-PEG to determine the cytotoxic effects of the polymers. After treatment, the media were removed and replaced with 20 μL of MTS reagent and 100 μL of culture media. They were left to incubate at 37 °C for 2 h in a 5% CO_2_ incubator. Measurement was then taken at 490 nm with a microplate reader. GraphPad Prism5 software was utilized to generate the IC_50_ value.

### 4.7. Statistical Analysis

Statistical analysis was carried out using GraphPad Prism5 software (San Diego, CA, USA). Each experiment was completed in triplicate and represented as mean ± standard error of the mean (SE). Comparison between means of two groups was made with *t*-test analysis.

## 5. Conclusions

This research presents a proof-of-concept for TQ encapsulating with polymeric PLGA-PEG and Pluronics F68 using a simple emulsion–solvent evaporation technique. This study has successfully demonstrated (1) synthesizing TQ-PLGA-PF68 nanoparticles that are <100 nm in size; (2) physicochemical properties and morphology of TQ-PLGA-PF68 nanoparticles that would support a better interaction between the nanoparticles and the target sites; (3) in vitro cytotoxicity evaluation of TQ-PLGA-PF68 nanoparticles to parental and resistant breast cancer cells that exert as a promising nanocarrier.

## Figures and Tables

**Table 1:** FTIR analysis of functional groups present in compounds studied.

Functional Group	Type of Compounds			
	TQ	Pluronics F68	PLGA-PEG	TQ-PLGA-PF68
sp^2^ CH	3257 (w)	-	-	-
sp^3^ CH	2967 (m)	2882 (m)	-	2882 (m)
N-H	-	-	2954 (w)	3345 (m)
C=C	1645 (s)	-	-	-
C=O	-	-	1748 (s)	1751 (m)
C=CH	3039 (w)	-	-	-
C-O-C	-	1100 (s); 1242 (m)	-	1103 (s)
C-O	-	-	1085 (s); 1127(s); 1164 (s)	-

w = weak; m = medium; s = strong.

**Table 2:** Resistant Index (RI) of different breast cancer subtypes after treatment with the anti-cancer drug and RI classification.

Resistant Cell Line	Drug	RI	* *p*-Value	RI Classification
TamR MCF-7	Tamoxifen	2.32	<0.05	Moderately drug- resistant
PacR MDA-MB- 231	Paclitaxel	1.59	<0.05	Drug-resistant
TamR UACC 732	Tamoxifen	1.32	<0.05	Drug-sensitive

RI: 0–2 = drug sensitive; 2–10 = moderately drug-resistant; >10 = drug-resistant [30,31]. * *t*-test analysis, n = 3.

**Table 3:** Comparison of IC_50_ value between the parental and chemoresistant cells.

A Subtype of Breast Cancer Cells	IC_50_ Value (Mean ± SE)		
	Parental Cell Line	Resistant Cell Line	* *p*-Value
MCF-7	90.46 ± 22.63	96.34 ± 1.70	> 0.05
UACC 732	118.5 ± 23.49	14.81 ± 3.2	<0.05
MDA-MB 231	34.28 ± 11.81	28.89 ± 5.12	<0.05

* *t*-test analysis, mean ± SE. n = 3 independent experiments.

## Data Availability

The data presented in this study are openly available in Figshare doi.org/10.6084/m9. figshare.16529781.v2 and doi.org/10.6084/m9.figshare.16529799.v1.
